# Impact of aging on calcium influx and potassium channel characteristics of T lymphocytes

**DOI:** 10.18632/oncotarget.3808

**Published:** 2015-04-12

**Authors:** Szonja Kollár, László Berta, Zsófia Eszter Vásárhelyi, Attila Balog, Barna Vásárhelyi, János Rigó, Gergely Toldi

**Affiliations:** ^1^ Second Department of Obstetrics and Gynecology, Semmelweis University, Budapest, Hungary; ^2^ First Department of Pediatrics, Semmelweis University, Budapest, Hungary; ^3^ First Department of Obstetrics and Gynecology, Semmelweis University, Budapest, Hungary; ^4^ Department of Rheumatology, University of Szeged, Szeged, Hungary; ^5^ Department of Laboratory Medicine, Semmelweis University, Budapest, Hungary; ^6^ MTA-SE Research Group of Pediatrics and Nephrology, Hungarian Academy of Sciences, Budapest, Hungary; ^7^ Bionics Innovation Center, Pázmány Péter Catholic University, Budapest, Hungary

**Keywords:** Kv1.3, IKCa1, Th2, CD8, immunosenescence

## Abstract

Adaptive immunity and T cell function are affected by aging. Calcium influx patterns, regulated by Kv1.3 and IKCa1 potassium channels, influence T cell activation. We aimed to compare calcium influx kinetics in CD8, Th1 and Th2 cells in human peripheral blood samples obtained from five different age groups (cord blood, 10-15 ys, 25-40 ys, 45-55 ys, 60-75 ys).

We measured calcium influx using flow cytometry in samples treated with or without specific inhibitors of Kv1.3 and IKCa1 channels (MGTX and TRAM, respectively).

Calcium influx was higher in Th1 cells of adults, however, its extent decreased again with aging. Importantly, these changes were not detected in Th2 cells, where the pattern of calcium influx kinetics is similar throughout all investigated age groups. MGTX had a more pronounced inhibitory effect on calcium influx in Th2 cells, while in Th1 cells the same was true for TRAM in the 25-40 ys and 45-55 ys groups. Calcium influx of CD8 cells were inhibited to a similar extent by both applied inhibitors in these groups, and had no effect in the elderly.

Altered lymphocyte potassium channel inhibitory patterns, regulators of calcium influx kinetics, might contribute to the development of age-related changes of T cell function.

## INTRODUCTION

Physiological functions of an individual are characterized by a decrease associated with aging in almost every organ system, and the immune system is no exception. Adaptive immunity is affected to a major extent by this process compared with the innate arm of immunity. Alterations of the aging immune system are of particular importance as they contribute to a higher incidence of infectious diseases and cancer, as well as to decreased efficacy of vaccinations observed in the elderly [1-3]. Among all cells with immunological functions, it is the T cells that are most altered. The most substantial age-related changes within the T cell compartment are a decrease in the number of antigen-inexperienced naive T lymphocytes and an increase in antigen-experienced memory and effector T cells [4]. This is in part due to a decrease in functional thymic mass with age and the consequent reduction in naive T cell output. Further effects of aging on the immune system are telomere shortening, changes in T cell signalling, along with impaired DNA repair and antioxidant mechanisms [5]. One of the earliest observations regarding T cells is their reduced IL-2 secretion and proliferation in aged individuals [6, 7], probably due to altered signalling.

Data from murine studies support an age-related shift from a Th1-like to a Th2-like cytokine response; however, whether or not this shift is characteristic in humans is not certain. A review of over 60 studies in humans suggests that age-associated changes in cytokine production are inconsistent [8]. These studies highlight that the stimulus used for the induction of cytokines in different studies influences both the level and pattern of immune response.

Declining T cell input affects functional subsets differentially [9]. It has been shown that with the progression of aging, Th1 cell numbers decline first, followed later by Th2 cells [10]. While aged naive CD4 T cells do not differentiate well to Th1 and Th2 effector subsets [11], they retain the ability to generate functional Th17 effectors. This is reflected by a greater prevalence of Th17 cells in the aged [12]. At the same time the percentage and function of regulatory T cells is also increased compared to young individuals [13]. However, the Th17/Treg ratio decreased age-dependently after stimulation and was accompanied by elevated FoxP3 mRNA and IL-10 protein expressions. Changes of the Th17/Treg ratio in combination with altered cytokine expression during aging may contribute to an imbalance between the pro-inflammatory and the anti-inflammatory immune response [14].

While changes in the prevalence of distinct T cell subsets have been well described, less is known on functional alterations affecting the short-term activation mechanisms of these subsets. During the activation of T cells a biphasic increase of the cytosolic calcium concentration is observed. During the first phase, the intracellular calcium stores located in the endoplasmic reticulum release calcium into the cytosol. During the second phase, the depletion of the intracellular stores evokes the opening of the calcium release activated calcium (CRAC) channels expressed in the cell membrane. These channels sustain elevated calcium levels via further entry of calcium from the extracellular space [15]. During this increase of calcium concentration, cell surface expressed potassium channels release potassium to maintain the negative membrane potential, hence further positively charged calcium ions may enter the cell, contributing to the maintenance of the later phase of T cell activation, including NFAT (nuclear factor of activated T cells) translocation into the nucleus and the following RNA transcription and cytokine production, respectively. There are two potassium channels which are important in this process: the voltage gated Kv1.3 and the calcium-dependent IKCa1 channels [16].

Recent results suggest that these channels represent potential therapeutic targets in autoimmune disorders, and their selective inhibition brought promising results in diseases such as rheumatoid arthritis and type one diabetes mellitus [17].

In this study, we aimed to compare calcium influx kinetics in CD8, Th1 and Th2 cells in human peripheral blood samples obtained from five different age groups ranging from cord blood to the elderly. We also aimed to study the age-characteristic differences evoked in calcium influx upon the application of specific inhibitors of lymphocyte potassium channels.

## RESULTS

**Figure 1:**
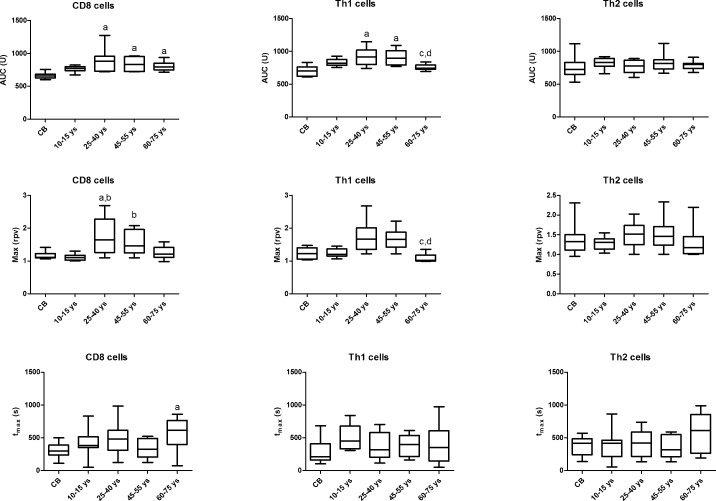
Area under the curve (AUC), Max and t_max_ parameter values of calcium influx in T cell subsets across the investigated age groups Horizontal line: median, box: interquartile range, whisker: range. ^a^ *vs.* CB, *p* < 0.05; ^b^ *vs.* 10-15 ys, *p* < 0.05; ^c^ *vs.* 25-40 ys, *p* < 0.05; ^d^ *vs.* 45-55 ys, *p* < 0.05; CB – cord blood.

Upon T cell activation, AUC values were higher in CD8 cells in the 25-40 ys, 45-55 ys and 60-75 ys age groups compared to CB and the 10-15 ys age group. Max values were also higher in the 25-40 ys and 45-55 ys age groups compared to the 10-15 ys group. AUC values were higher in Th1 cells in the 25-40 ys and 45-55 ys age groups and decreased again in the 60-75 ys group to the levels observed in CB and in the 10-15 ys group. Max values were also decreased in the 60-75 ys group compared to the 25-40 ys and 45-55 ys age groups. No difference was observed in these values in the Th2 subset across the investigated age groups (Figure 1).

**Figure 2:**
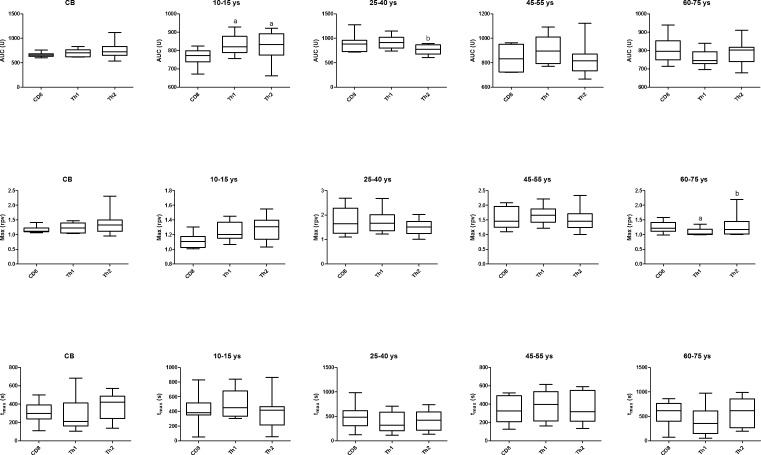
Area under the curve (AUC), Max and t_max_ parameter values of calcium influx in the investigated age groups in CD8, Th1 and Th2 cells Horizontal line: median, box: interquartile range, whisker: range. ^a^ *vs.* CD8, *p* < 0.05; ^b^ *vs.* Th1, *p* < 0.05; CB – cord blood.

AUC values of Th1 and Th2 cells were higher than that of CD8 cells in the 10-15 ys age group. In the 25-40 ys group AUC of Th2 cells was lower than that of Th1 cells. Max value of Th1 cells was lower in the 60-75 ys group than that of CD8 or Th2 cells (Figure 2).

Treatment with specific inhibitors of the Kv1.3 and IKCa1 channels had no effect on calcium influx parameters on CB samples. AUC of the Th1 subset was decreased by TRAM to a greater extent than by MGTX in the 10-15 ys, 25-40 ys and 45-55 ys groups. On the contrary, AUC or Max values of the Th2 subset were decreased to a greater extent by MGTX in these age groups. t_max_ was decreased by MGTX in Th2 cells of the 60-75 ys group. No other effect of the inhibitors was observed in this age group (Table 1).

**Table 1:** The effects of margatoxin (MGTX) and triarylmethane (TRAM) on calcium influx of T cell subsets

	CD8 AUC (U)	CD8 Max (rpv)	CD8t_max_ (s)	Th1 AUC (U)	Th1 Max (rpv)	Th1 t_max_ (s)	Th2 AUC (U)	Th2 Max (rpv)	Th2 t_max_ (s)
CB no inhibitor	656 [602-758]	1.117 [1.066-1.414]	297 [110-500]	705 [613-831]	1.225 [1.037-1.473]	209 [105-683]	724 [532-1117]	1.329 [1.003-2.311]	419 [138-570]
CB MGTX treated CB TRAM treated	619 [563-721]	1.086 [1.005-1.305]	257 [102-486]	645 [593-765]	1.220 [1.009-1.542]	181 [88-457]	690 [475-878]	1.323 [1.002-1.873]	279 [12-578]
	602 [570-712]	1.042 [1.002-1.315]	211 [66-570]	643 [598-775]	1.175 [1.018-1.477]	148 [75-215]	642 [515-1019]	1.207 [1.003-2.016]	270 [200-869]
10-15 ys no inhibitor	773 [739-798]	1.106 [1.026-1.174]	381 [353-515]	820 [790-877]	1.202 [1.151-1.369]	451 [334-679]	833 [775-891]	1.308 [1.138-1.396]	417 [214-465]
10-15 ys MGTX treated 10-15 ys TRAM treated	754 [742-831]	1.104 [1.031-1.274]	695* [490-832]	807 [776-818]	1.148 [1.086-1.209]	556 [348-735]	788 [774-810]	1.106* [1.091-1.154]	565 [441-806]
	769 [730-795]	1.084 [1.041-1.193]	490 [259-540]	745* [725-768]	1.035 [1.002-1.173]	382 [110-594]	794 [759-839]	1.404 [1.131-2.164]	501 [300-658]
25-40 ys no inhibitor	831 [727-950]	1.462 [1.260-2.041]	326 [205-478]	868 [781-1002]	1.588 [1.333-1.931]	353 [227-486]	777 [752-854]	1.460 [1.258-1.546]	393 [228-536]
25-40 ys MGTX treated 25-40 ys TRAM treated	760* [707-923]	1.558* [1.245-1.804]	325 [211-439]	881 [707-907]	1.555* [1.336-1.699]	373 [216-542]	656* [642-782]	1.237* [1.122-1.411]	414 [258-641]
	780* [665-987]	1.437 [1.181-1.940]	234 [165-422]	797* [769-852]	1.547 [1.415-1.735]	532 [333-570]	694* [654-782]	1.144* [1.115-1.549]	457 [336-532]
45-55 ys no inhibitor	831 [742-950]	1.462 [1.260-1.918]	326 [253-478]	897 [805-1006]	1.660 [1.466-1.846]	396 [227-515]	790 [751-857]	1.459 [1.249-1.574]	365 [214-537]
45-55 ys MGTX treated 45-55 ys TRAM treated	735* [700-873]	1.305* [1.235-1.605]	317 [227-336]	855* [703-915]	1.538* [1.299-1.617]	339 [214-512]	655* [643-726]	1.141* [1.096-1.239]	352 [231-498]
	722* [659-780]	1.302* [1.134-1.429]	309 [204-457]	761* [724-794]	1.431* [1.348-1.633]	460 [314-549]	699* [654-777]	1.200* [1.120-1.479]	438 [346-515]
60-75 ys no inhibitor	796 [750-853]	1.214 [1.112-1.417]	615 [398-764]	745 [728-792]	1.025 [1.001-1.180]	353 [147-608]	802 [740-817]	1.176 [1.020-1.454]	613 [264-856]
60-75 ys MGTX treated	793 [771-865]	1.168 [1.106-1.273]	470 [272-643]	774 [757-799]	1.111 [1.046-1.160]	453 [259-585]	760 [724-792]	1.143 [1.044-1.416]	269* [97-532]
60-75 ys TRAM treated	807 [775-937]	1.270 [1.113-1.520]	418 [131-736]	774 [738-831]	1.133 [1.071-1.411]	640 [413-750]	752 [737-794]	1.113 [1.002-1.183]	409 [133-573]

Data are expressed as median [IQR].

* vs. no inhibitor sample in the same age group, p < 0.05. CB – cord blood, AUC – area under the curve

## DISCUSSION

The immune system is affected by aging and undergoes significant age-related changes, commonly termed immunosenescence. Different T cell subsets are affected to different extent by this process. Earlier studies described the alterationsoCcurring in the prevalence of certain T cell subpopulations during aging. Although data on the proportion of T helper subsets in the aged individual are not fully consistent, a decrease in Th1 along with an increase in Th17 cell numbers has been observed [9, 10, 13]. While changes affecting T cell percentages are of great interest, functional alterations, especially those of intracellular signalling mechanisms, are equally important but less studied during immunosenescence. As noted before, one of the earliest observations regarding T cells is their reduced IL-2 secretion and proliferation in aged individuals [6, 7], probably due to altered signalling.

In our study we aimed to describe alterations affecting the initial calcium response following TCR stimulation in CD8, Th1 and Th2 cells. We found that calcium influx was higher in adults, including the elderly, than in CB and children in case of CD8 cells. The peak of cytoplasmic calcium concentration was reached later specifically in CD8 cells in the elderly, indicating that signal transduction might take longer in this subset in aging. Calcium influx was also higher in Th1 cells of adults compared to CB and children, however, its extent decreased again with aging. Importantly, these changes were not detected in Th2 cells, where the pattern of calcium influx kinetics is similar throughout all investigated age groups.

Calcium influx was higher in CD4 than in CD8 cells in children. In young adults, Th2 cells responded with lower calcium flux compared to Th1 cells upon the same activating stimulus. This is in line with earlier observations of Fanger et al. Using fura-2 calcium imaging of murine Th1 and Th2 clones, they observed that the calcium rise elicited following store depletion with thapsigargin is significantly lower in Th2 cells than in Th1 cells [18]. On the contrary, the peak cytoplasmic calcium concentration was higher in the Th2 subset compared to Th1 cells in the elderly.

Lymphocyte potassium channel inhibition had no effect in CB. The only effect observed in the elderly was that the peak of calcium influx was reached earlier in Th2 cells upon the inhibition of Kv1.3 channels. Calcium influx was decreased by TRAM, the blocker of IKCa1 channels in Th1 cells of children, while MGTX decreased the peak cytoplasmic calcium concentration in Th2 cells in this age group. Similarly, MGTX had a more pronounced inhibitory effect on calcium influx in Th2 cells, while TRAM had a more pronounced inhibitory effect in Th1 cells in both adult groups. Calcium influx of CD8 cells were inhibited to a similar extent by both applied inhibitors in the two adult groups, and had no effect in the elderly. This characteristic pattern of lymphocyte potassium channel function, important regulators of calcium influx kinetics, seems to develop with aging into adulthood, but disappears in the elderly, probably contributing to the development of alterations playing a role in immunosenescence.

One of the major defects in the responsiveness of aged naive CD4 cells has been shown to be owing to the reduced ability of these cells to form highly functional immunological synapses upon stimulation with peptide antigen and antigen presenting cells [19]. Because of reduced synapse formation, the initial signalling cascades generated in the aged naive CD4 cells are less intense than those in young T cells. This then contributes to the well-documented reduced proliferation of CD4 cells. The ultimate result of this defect is that aged naive CD4 cells do not expand, produce cytokines, and differentiate as well as those from the young.

Our findings might highlight the fact that altered calcium influx might be part of this phenomenon, but affects distinct T helper subsets (and cytotoxic T cell) to a different extent, with a more pronounced decrease in Th1 cells.

Gupta demonstrated that the basal cytoplasmic calcium levels in aging T cells were lower than that in young T cells, and a much smaller rise in cytoplasmic calcium was observed following activation with PHA and anti-CD3 monoclonal antibody in aging T cells than in the young T cells [20]. In this study, distinct T cell subsets were not distinguished, and taking into account our current results it seems that this observation is in line only in case of Th1 cells, since the rise of cytoplasmic calcium was comparable to young adults in case of Th2 and CD8 cells in our study.

Further alterations with aging that have been described in T cell signalling include changes of protein kinase C translocation [21], NFAT distribution [22], Lck activation [23], linker of activated T cell activation [24], Fyn activation [22] and ZAP-70 activation [25], all of which might be influenced by the altered calcium influx signalling.

Earlier results suggested that advancing age is a risk factor for autoimmunity and is associated with chronic inflammation [26]. Our results of decreased calcium influx in Th1 cells with aging do not support this notion. This is also reinforced by the clinical observation that although the prevalence of some autoantibodies is increasing with age, the frequency of new incidences of autoimmune diseases is not following [27].

In summary, calcium influx was higher in adults in CD8 cells. Calcium influx was also higher in Th1 cells of adults compared to CB and children, however, its extent decreased again with aging. Importantly, these changes were not detected in Th2 cells, where the pattern of calcium influx kinetics is similar throughout all investigated age groups. MGTX had a more pronounced inhibitory effect on calcium influx in Th2 cells, while in Th1 cells the same was true for TRAM in both adult groups. Calcium influx of CD8 cells were inhibited to a similar extent by both applied inhibitors in the two adult groups, and had no effect in the elderly. This characteristic pattern of lymphocyte potassium channel function, important regulators of calcium influx kinetics, seems to develop with aging into adulthood, but disappears in the elderly, probably contributing to the development of alterations playing a role in immunosenescence.

## MATERIALS AND METHODS

### Sample collection

Cord blood samples were collected from 9 healthy, term neonates (5 girls and 4 boys, gestational age: 40 (38-41) weeks, median (range), birth weight: 3450 (3050-3900) grams, median (range)). Peripheral blood samples were taken from 9 healthy children (4 girls and 5 boys, age: 13 (10-15) years, median (range)), 10 young adults (5 women and 5 men, age: 35 (25-40) years, median (range)), 10 middle-aged adults (5 women and 5 men, age: 49 (45-55) years, median (range)), and 12 elderly volunteers (7 women and 5 men, age: 67 (60-75) years, median (range)). All participating adults fulfilled the criteria of the SENIEUR protocol [28]. Informed consent was obtained from all subjects, or in case of minors, parents of all subjects, and our study was reviewed and approved by an independent ethical committee of the institution. The study was adhered to the tenets of the most recent revision of the Declaration of Helsinki.

### Calcium influx measurements

Cord and peripheral blood mononuclear cells (CBMCs and PBMCs) were separated by a standard density gradient centrifugation (25 min, 400 *g*, room temperature) from freshly drawn blood. This cell suspension was washed twice in PBS. From then on, cells were kept throughout staining with fluorescent markers, treatment with inhibitors and measurement on flow cytometer in a modified RPMI-1640 medium. The calcium concentration of this medium was set to 2 mM by addition of crystalline CaCl_2_.

PBMCs were incubated with the following anti-human mAbs: anti-CD4 PE-Cy7, anti-CXCR3 APC, anti-CCR4 PE and anti-CD8 APC-Cy7 (all from BD Biosciences, San Jose, CA, USA) according to the manufacturer's instructions. Cytoplasmic free calcium levels were detected by loading the cells with the 1:2 ratio of Fluo-3-AM and Fura Red fluorescent dyes and 0.02 % Pluronic F-127 (Molecular Probes, Karlsbad, CA, USA) for 20 minutes at 30°C. Cells were washed once before measurement.

PBMCs were divided into three vials with equal cell numbers. One vial was treated with margatoxin (MGTX, 4 nM; Sigma-Aldrich, St. Louis, MO, USA), a selective blocker of the Kv1.3 channel, for 15 min before measurement. Another vial was treated with a triarylmethane compound, TRAM-34 (240 nM; Sigma-Aldrich), a specific inhibitor of the IKCa1 channel, for 10 min before measurement. The third vial was used as control. In all cases, samples were kept at 30°C in dry bath, until the measurements.

T cell activation was initiated by phytohemagglutinin (15 μg/ml final concentration). Fluorescence emission of sequentially measured cells was monitored for 10 minutes by flow cytometry. Average cell acquisition rate was 1000 cells/s. All measurements were performed on a BD FACSAria flow cytometer (BD Biosciences). The population of lymphocytes was gated according to forward and side scatter characteristics. CD4+ CXCR3+ cells were regarded as Th1 cells, CD4+ CCR4+ cells were regarded as Th2 cells, CD8+ cells were regarded as the cytotoxic T cell population.

Data acquired from the measurements were evaluated with specific software developed at our laboratory. The core of this software is an algorithm [29] based on the calculation of double-logistic functions for each recording. The software also calculates parameter values describing each function, such as the area under the curve (AUC) value, corresponding to the sum of the cytoplasmic calcium increase, the maximum value, representing the peak cytoplasmic calcium concentration during activation, and the time to reach maximum value.

Data are expressed as median [interquartile range].

Comparisons of the calculated parameters between T cell subpopulations in the same patient group as well as between patient groups within the same T cell subset were made with the Kurskall-Wallis test, while the effect of the applied channel blockers were calculated using the Wilcoxon-test, as Kolmogorov–Smirnoff analysis indicated non-normal distribution of data. Correlation analysis of the clinical and laboratory parameters with the calcium influx values were executed by Spearman rank correlation. Statistics were calculated at 5% significance level (p = 0.05) using the GraphPad Prism 5 software (La Jolla, CA, USA).
